# Identifying Predictors of Initial Surgical Failure during Minimally Invasive Endoscopic Intracerebral Hemorrhage Evacuation

**DOI:** 10.3390/biomedicines12030508

**Published:** 2024-02-23

**Authors:** Turner S. Baker, Roshini Kalagara, Ayesha Hashmi, Benjamin Rodriguez, Shelley H. Liu, Hana Mobasseri, Colton Smith, Benjamin Rapoport, Anthony Costa, Christopher P. Kellner

**Affiliations:** 1 Sinai BioDesign, Icahn School of Medicine at Mount Sinai, New York, NY 10029, USA; roshini.kalagara@icahn.mssm.edu (R.K.); ayesha.hashmi@icahn.mssm.edu (A.H.); benjamin.rodriguez@icahn.mssm.edu (B.R.); anthony.costa@gmail.com (A.C.); 2 Department of Neurosurgery, Icahn School of Medicine at Mount Sinai, New York, NY 10029, USA; colton.smith@mountsinai.org (C.S.); christopher.kellner@mountsinai.org (C.P.K.); 3 Department of Population Health Science and Policy, Icahn School of Medicine at Mount Sinai, New York, NY 10029, USA; shelley.liu@mountsinai.org (S.H.L.); hana.mobasseri@icahn.mssm.edu (H.M.)

**Keywords:** intracerebral hemorrhage, intraoperative MRI, stroke, minimally invasive surgery, hemorrhage

## Abstract

**Background and Purpose**: Intracerebral hemorrhage (ICH) is a common and severe disease with high rates of morbidity and mortality; however, minimally invasive surgical (MIS) hematoma evacuation represents a promising avenue for treatment. In February of 2019, the MISTIE III study found that stereotactic thrombolysis with catheter drainage did not benefit patients with supratentorial spontaneous ICH but that a clinical benefit may be present when no more than 15 mL of hematoma remains at the end of treatment. Intraoperative CT (iCT) imaging has the ability to assess whether or not this surgical goal has been met in real time, allowing for operations to add additional CT-informed ‘evacuation periods’ (EPs) to achieve the surgical goal. Here, we report on the frequency and predictors of initial surgical failure on at least one iCT requiring additional EPs in a large cohort of patients undergoing endoscopic minimally invasive ICH evacuation with the SCUBA technique. **Methods**: All patients who underwent minimally invasive endoscopic evacuation of supratentorial spontaneous ICH in a major health system between December 2015 and October 2018 were included in this study. Patient demographics, clinical and radiographic features, procedural details, and outcomes were analyzed retrospectively from a prospectively collected database. Procedures were characterized as initially successful when the first iCT demonstrated that surgical success had been achieved and initially unsuccessful when the surgical goal was not achieved, and additional EPs were performed. The surgical goal was prospectively identified in December of 2015 as leaving no more than 20% of the preoperative hematoma volume at the end of the procedure. Descriptive statistics and regression analyses were performed to identify predictors of initial failure and secondary rescue. **Results**: Patients (100) underwent minimally invasive endoscopic ICH evacuation in the angiography suite during the study time period. In 14 cases, the surgical goal was not met on the first iCT and multiple Eps were performed; in 10 cases the surgical goal was not met, and no additional EPs were performed. In 14 cases, the surgical goal was never achieved. When additional EPs were performed, a rescue rate of 71.4% (10/14) was seen, bringing the total percentage of cases meeting the surgical goal to 86% across the entire cohort. Cases in which the surgical goal was not achieved were significantly associated with older patients (68 years vs. 60 years; *p* = 0.0197) and higher rates of intraventricular hemorrhage (34.2% vs. 70.8%; *p* = 0.0021). Cases in which the surgical goal was rescued from initial failure had similar levels of IVH, suggesting that these additional complexities can be overcome with the use of additional iCT-informed EPs. **Conclusions**: Initial and ultimate surgical failure occurs in a small percentage of patients undergoing minimally invasive endoscopic ICH evacuation. The use of intraoperative imaging provides an opportunity to evaluate whether or not the surgical goal has been achieved, and to continue the procedure if the surgeon feels that more evacuation is achievable. Now that level-one evidence exists to target a surgical evacuation goal during minimally invasive ICH evacuation, intraoperative imaging, such as iCT, plays an important role in aiding the surgical team to achieve the surgical goal.

## 1. Introduction

Spontaneous intracerebral hemorrhage (ICH) is a common and devastating disease with an estimated annual worldwide incidence of 16 per 100,000 and a US incidence of 26 per 100,000, a number that is expected to increase with the increasing age of the population [1,2,3,4]. ICH-related mortality is high, and just 20% of patients achieve functional independence 6 months following the hemorrhage [1]. Conventional surgical approaches to treat ICH have shown mixed results, with multiple studies demonstrating a lack of functional benefits for patients undergoing surgery and others showing only a modest improvement in outcomes [5,6]. Much of the field has shifted focus to minimally invasive surgical (MIS) interventions [7], resulting in the development of various promising technical approaches [8]. Minimally invasive ICH evacuation has been studied in numerous clinical trials, with a recent study-level meta-analysis and systematic review, suggesting an overall benefit to the procedure in the odds of reducing the chance of dependency and death [9,10].

The MISTIE III trial, however, recently found no improvement in long-term functional outcome following minimally invasive catheter drainage with thrombolysis compared to standard medical management in a highly selected cohort [8]. In a prespecified as-treated analysis, however, investigators showed that hematoma evacuation percentage correlated with long-term functional improvement with overall benefit compared to medical management reached in the cohort of patients in whom the surgical goal of leaving no more than 15 mL at the end of treatment was achieved. This analysis suggests that MIS ICH evacuation could be beneficial if sufficient evacuation can be consistently and safely performed [8]. 

In December of 2015, the Mount Sinai Health System implemented an institutional protocol to perform minimally invasive endoscopic ICH evacuation for patients with prospectively developed inclusion and exclusion criteria based on the available literature with a predefined surgical goal of removing at least 80% of the hematoma. This procedure is performed in the angiography suite (angiosuite), permitting the use of intraoperative conebeam CT (iCT) after evacuation and prior to the completion of the procedure. When a procedure fails to meet the surgical goal, >80% evacuation, the surgeon can continue into another ‘evacuation period’ (EP) in order to achieve the surgical goal. A procedure is considered ‘rescued’ when the initial iCT is found to miss the surgical goal, but further evacuation is performed eventually achieving the surgical goal. 

In this study, we aim to evaluate predictors of surgical goal achievement for patients undergoing minimally invasive surgical (MIS) hematoma evacuation. We hypothesize that the usage of intraoperative CT will allow for more consistent operative success.

## 2. Material and Methods

### 2.1. Study Design

The study was a retrospective analysis of prospectively collected data from a single-center observational cohort. The Mount Sinai Institutional Review Board approved all protocols and analyses (IRB-19-01479).

### 2.2. Patient Population

All patients in this study presented to the Mount Sinai Health System between December 2015 and October 2018 with a spontaneous ICH, and were evaluated for treatment with minimally invasive endoscopic ICH evacuation using the Stereotactic Underwater Blood Aspiration (SCUBA) technique and triaged to the hospital’s dedicated ICH program. The technical details of this procedure [11] and inclusion and exclusion criteria for this cohort have been previously described [12]. The prospectively determined surgical goal was to remove at least 20% of the preoperative hematoma as assessed using the ABC/2 method of the ICH volume calculation on the conebeam intraoperative CT (iCT) images in real-time. iCT was performed at least once in all procedures prior to closure. If the surgical goal had not been achieved, the operating surgeon then decided if further evacuation was safe and feasible. If deemed safe and feasible, one or more additional passes were then made with iCT occurring after each evacuation period to re-evaluate if the surgical goal had been achieved.

One-hundred patients who underwent MIS-ICH evacuation during the study period were included. Data were extracted from a prospectively collected quality assurance database. Patient variables collected included age, gender, premorbid baseline hypertension (HTN), anticoagulant-use, antiplatelet-use; clinical variables including baseline modified Rankin Score (mRS), ICH score, NIH Stroke Score (NIHSS); radiographic variables including hematoma location, intraventricular hemorrhage (IVH), spot-sign, preoperative hematoma volume, and postoperative hematoma volume; procedural details including bleed-to-evacuation time, admission-to-evacuation time, and procedure time; and outcomes including neurosurgical intensive care unit length of stay (NSICU LOS), hospital length of stay (LOS), discharge location, 1-month motility, and 6-month mRS. An intraoperative conebeam CT was used in all procedures to assess the degree of hematoma evacuation. Intraoperatively, residual hematoma was estimated using the ABC/2 method.

### 2.3. Statistical Methods

The dataset was dichotomized by cases that achieved the surgical goal at the end of the first EP, where the initial iCT showed ≥80% reduction in hematoma size compared to the preoperative volumetric analysis (Table 1). The cohort of cases that had undergone multiple EPs was then dichotomized into two groups, based on those that ultimately achieved the surgical goal and those that failed to ever achieve ≥80% evacuation (Table 2). Postoperative rebleeds where bleeding occurred after the conclusion of the surgery were considered to have successfully achieved the surgical goal, and all references to ‘postoperative volume’ refer to intraoperative volumetric assessment. Descriptive analysis was performed to compute the frequencies and percentages of key patient characteristics and surgical elements. Statistically significant differences were tested using Fisher’s exact test for categorical variables and non-parametric Wilcoxon rank sum test for continuous variables (Table 1 and Table 2). To assess the association of additional EPs on total surgical time, a univariate and multivariate linear regression model were selected. A 2-sided *p* = 0.05 was used to indicate statistical significance. Descriptive statistics, analysis and regression models were calculated using SAS software (SAS Institute Inc., Cary, NC, USA, https://www.sas.com/en_us/home.html).

## 3. Results

**Figure 1:**
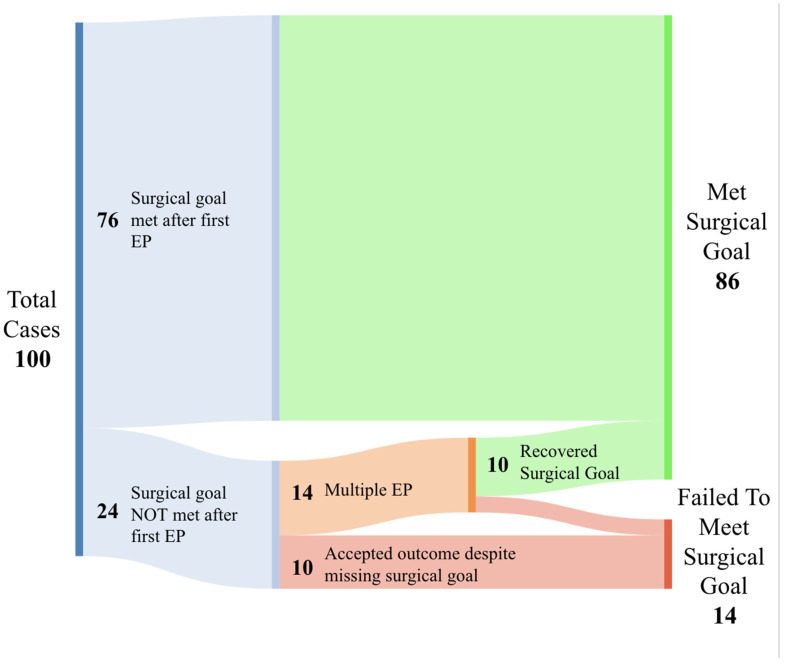
Sankey diagram outlining the distribution of failed, rescued and successful cases. Green indicates cases that were able to achieve surgical goals, orange indicates cases that used multiple EPs to attempt to achieve the surgical goal, and red indicates cases that failed to achieve the surgical goal.

The surgical goal was achieved at the end of the first EP, confirmed through an iCT, in 76% (76/100) of cases (Figure 1). Of the 24% (24/100) remaining, 14 cases initiated an additional EP. Cases where an additional EP was performed following the initial iCT resulted in a rescue rate of 71.4% (10/14), bringing the total percentage of cases meeting the surgical goal to 86% across the entire cohort. Moreover, rebleeds occurred in five cases, with 80% (*n* = 4) being within 24 h of the initial procedure. Re-evacuation was required for one of these cases. 

**Figure 2:**
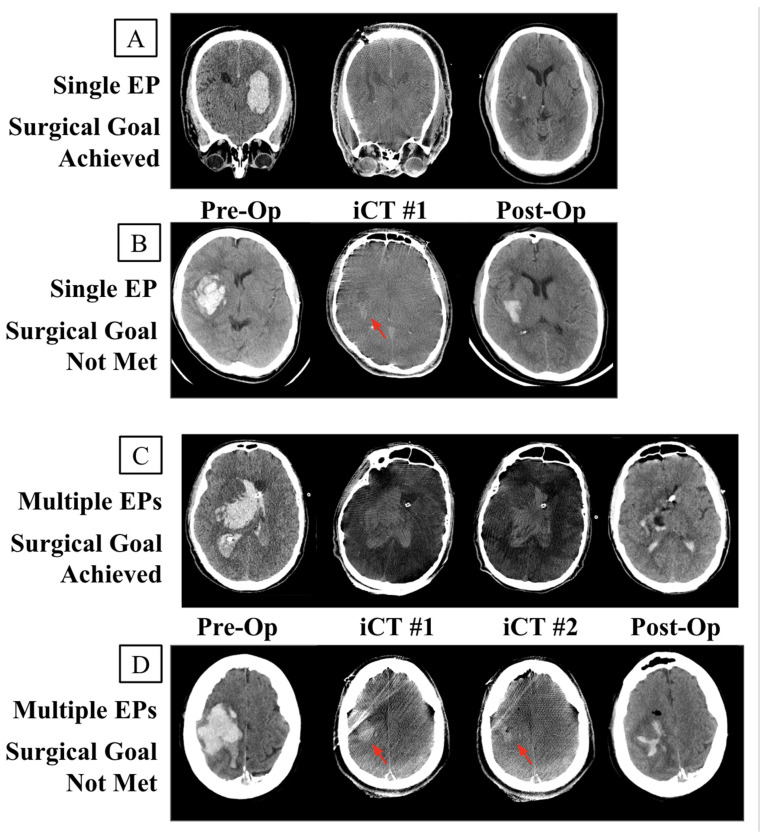
Intraoperative (iCT) and postoperative CT of each case type. (**A**) Successful surgical goal achieved upon the completion of the original EP. (**B**) Surgical goal not achieved but no secondary EPs conducted due to additional complications. (**C**) Surgical goal not achieved at the time of the initial iCT but achieved following multiple EPs. (**D**) Multiple EPs attempted to rescue the surgical goal without success. Red arrow identifying residual bleed.

In 10% (10/100) of cases, the initial iCT demonstrated a failure to meet the surgical goal, but no additional Eps were performed to attempt rescue (Figure 2B). 

**Figure 3:**
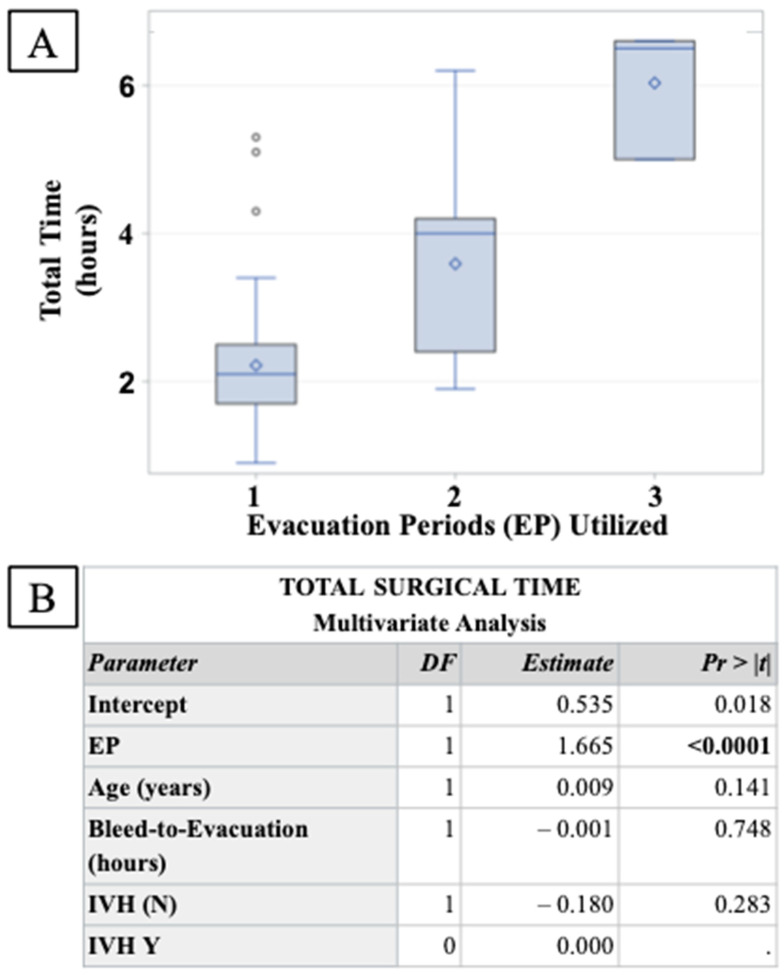
Assessment of surgical time increase in response to additional EPs. (**A**) A three-way ANOVA demonstrated a significant increase in total surgical time in response to additional EPs. (**B**) Multivariate linear regression analysis controlling for significant covariates found EP to be significantly associated with a 1.65 h increase in surgical time for each additional EP performed.

Primary analysis of the dataset was between groups that achieved the surgical goal at the completion of the first EP (*n* = 76) and those that had >20% residual hematoma at the time of the first iCT (*n* = 24) (Table 1). The group that failed to meet the surgical goal was found to be older (68 years vs. 60 years; *p* = 0.0197), and have a significantly increased proportion of intraventricular hemorrhage (IVH) (34.2% vs. 70.8%; *p* = 0.0021). Bleed-to-evacuation time was also found to be significantly shorter in failed cases (28 h vs. 41.25 h; *p* = 0.013). Multivariate logistic regression analysis was then used to control for all other covariates (Figure 3). Failure to achieve the surgical goal at the completion of the initial EP were 4.45 times more likely when a case presented with IVH, and increased with patient age by 5% per year. An increase in the bleed-to-evacuation time was found to be associated with a 3% reduction in initial surgical failure per hour (see Table 3). No significant interaction terms were identified. 

The 14 cases that failed to meet the surgical goal and underwent additional EPs to attempt to rescue the surgical goal were further stratified by success (*n* = 10, Figure 2C) or failure (*n* = 14, Figure 2D) (Table 2). A total of four cases where additional EPs were performed following the initial iCT did not result in the successful rescue of the surgical goal (Figure 2D). Bleed-to-evacuation times were again found to be significantly shorter in cases that failed to ultimately achieve the surgical goal. By design, postoperative hematoma volumes were found to be significantly lower in successful surgeries. No other significant group differences were identified.

To assess the surgical burden of additional EPs, a three-way ANOVA (Figure 3B) was performed to determine changes in total surgical time between each EP group. The procedural time was significantly larger with each additional EP, increasing total surgical time by 1.67 h for every additional EP performed while controlling for potentially confounding covariates.

## 4. Discussion

The results of this study demonstrate the complexity of MIS-ICH evacuation, and support the use of intraoperative imaging to ensure that a majority of cases achieve predetermined surgical outcomes. The ability to quantify the residual hematoma while the patient is still on the table is a useful and necessary tool, and clearly allows for complex cases to be rescued from incomplete evacuations. The concept of reporting on MIS-ICH evacuation with specified EPs separated by iCTs is novel, and should become a standardized reporting characteristic of evacuations performed with the assistance of intraoperative imaging. Through the use of iCT, the surgical goal was met in an additional 10 cases in this report.

IVH, age, and bleed-to-evacuation times were all found to affect the likelihood of a surgical failure at the time of the first iCT. Cases that failed to achieve the initial surgical goal were associated with a greater frequency of IVH, yet existed in equal proportion between multiple-EP cases that achieved or failed surgical goals. This suggests that the use of additional iCTs can assist surgeons in real time to navigate the added complexity of IVH in order to achieve evacuation targets. Older patients were found to be associated with greater surgical failure rates, potentially explained by the increased complexity of ICH evacuation caused by an amyloid buildup and resulting vessel fragility, which has previously been well established as a risk factor for ICH [13]. Unsurprisingly, the use of multiple EPs was associated with longer operational times, as the choice to continue towards surgical goals meant additional procedural time. Neither the evacuation percentage nor use of multiple EPs showed a significant effect in predicting postoperative clinical outcomes, however. Interestingly, prior literature analyzing operative duration and outcomes have generally noted increased complication risk with prolonged operative time, in both disease-specific studies and in a meta-analysis across several surgical specialties and procedure types [14,15,16]. As this effect was not seen prior, there may be a protective effect of the achieving a higher evacuation percentage that may bypass the potential pitfalls of increased operative duration.

There was no clear correlation between the ability of the surgeon to achieve high levels of evacuation and the preoperative volume of the bleed. This is likely due to ‘surgical success’ being defined as >80% evacuation, resulting in a bias away from cases with small initial bleeds that could leave minimal residual hematoma while still missing the <20% goal. Much of the field has already begun to use residual hematoma volume as a surgical success metric. In order to accurately illustrate the target of the surgeon at the time of surgery, the analysis of this study was conducted using the surgical target defined prior at trial initiation. The average evacuation percentage across the entire cohort (90.9%) was found to be slightly higher than the average evacuation percentage in the recent MISTIE III trial (69%) [8,17].

Overall, the results of this study demonstrate the value of performing MIS-evacuation of ICH in the angiosuite and for the use of iCT to assist surgeons during surgery. The growing data in support of ICH evacuation are promising; however, the lack of a conclusive study suggests that further refinement of the surgical protocol is needed. While increasing the experience and expertise of surgeons participating in clinical trials studying MIS-ICH evacuation will eventually lead to an improved evacuation rate, the potential for additional intraoperative imaging is primed to minimize this burden. In fact, this method of practice may be of special interest at teaching institutions with trainees that have varying backgrounds and levels of expertise, from residents to fellows. By incorporating a robust way of ensuring more standardized practices, intraoperative CT provides an opportunity for early-stage trainees to have more autonomy in surgical procedures, with real-time feedback and opportunities to improve operative techniques. This can translate into centers being able to increase surgical accuracy and volumes with more robustly trained trainees. 

Going forward, it will be essential for trials and standard practice to minimize residual hematoma and ensure ample evacuation prior to the end of the operation. Cases with increased complexity, such as large bleeds with IVH, are frequent enough to include in trials, and particularly benefit from surgeons having access to intraoperative imaging. The ability to perform iCTs within MIS-ICH evacuations clearly assists in accomplishing these goals and although there are current studies such as the present one discussing and evaluating the use of intraoperative imaging, there is a demonstrated need for standardized prospective trials [18,19,20].

This study suffers from several limitations, namely that the cohort contains surgeries from a single institution, limiting the external reliability of the findings. As more surgeons begin to perform this surgery in the angiosuite, larger, multi-site studies should be conducted. In addition, although patients were identified from a prospectively collected database, details relating to patient demographics, clinical symptoms, radiographic findings, procedural details, and outcomes were retrospectively abstracted and analyzed. Available information was limited to information that was found after review of the patient chart and more qualitative data were unable to be collected. As such, this limitation will be addressed by more robust prospective future trials. 

## 5. Conclusions

These findings demonstrate the potential benefit of intraoperative imaging during minimally invasive ICH evacuation to guide a surgeon in deciding whether or not to stop the procedure or continue the evacuation. While a majority of MIS-ICH evacuations can meet predefined surgical goals under standard procedures, this study demonstrated the use of intraoperative imaging to rescue low-evacuation surgeries by informing additional EPs. The use of iCT to achieve prespecified evacuation percentages and inform surgeons during complex cases, and the reporting of EPs required within a case will be critical to integrate into future protocols and practices.

## Figures and Tables

**Table 1:** Descriptive statistics between groups that achieved or failed surgical goals at the completion of the initial EP. Means and relative frequencies between case groups. Statistically significant differences were tested for using Fisher’s exact test for categorical variables and non-parametric Wilcoxon rank sum test for continuous variables. Significant *p* values on bold.

	Surgical Goal Not Achieved at First iCT (*n* = 24)	Surgical Goal Achieved at First iCT (*n* = 76)	*p*-Value
1.1 Patient Population Characteristics			
Age (years)	67.96	60.43	**0.0197**
Gender (%F)	29.2%	34.2%	0.8044
Race/Ethnicity			0.8129
Black	33.33	35.53	
Asian	16.67	18.42	
White	37.50	27.63	
Hispanic	12.50	18.42	
Baseline mRS	0.67	0.30	0.125
NIHSS	16.29	17.63	0.37
IVH (%Y)	70.8%	3421.0%	**0.0021**
HTN (%Y)	83.3%	79.0%	0.7747
Spot Sign (%Y)	20.8%	14.5%	0.5254
Brain Depth (%‘Deep’)	50.0%	67.1%	0.1506
1.2 Preoperative Care			
Bleed to Evacuation (hours)	28.04	41.25	**0.013**
Anticoagulants (%Y)	20.8%	7.9%	0.1271
Angiographic Event (%Y)	95.8%	84.2%	0.1804
Antiplatlets (%Y)	29.2%	27.6%	1
Pre-Op Volume (mL)	55.51	48.81	0.4502
1.3 Intraoperative Care			
Total Time (hours)	3.22	2.36	0.0619
1.4 Postoperative Outcomes			
Post-Op Volume (mL)	14.05	2.33	**0.0005**
Evacuation (%)	76.3%	95.5%	**0.0002**
NSICU LOS	9.58	11.30	0.3245
LOS	18.39	21.22	0.3805
6-month mRS	3.04	3.25	0.5766
Mortality (%Died)	8.3%	9.2%	1

mRS: modified Rankin Scale; NIHSS: NIH Stroke Scale; IVH: intraventricular hemorrhage; HTN: hypertension; NSICU LOS: neurosurgical intensive care unit length of stay; LOS: Length of stay.

**Table 2:** Descriptive statistics of multiple-EP cases: Means and relative frequencies were compared between cases that underwent multiple EPs and either achieved or failed the surgical goal of >80% evacuation at completion of the final EP. Significant *p* values on bold.

	Surgical Goal Missed after Multiple EPs (*n* = 4)	Surgical Goal Rescued after Multiple EPs (*n* = 10)	*p*-Value
1.1 Patient Population Characteristics			
Age (years)	63.75	64.00	0.9708
Gender (%F)	25.0%	20.0%	1
Race/Ethnicity			1
Black	25	40	
Asian	25	10	
White	25	30	
Hispanic	0	20	
Baseline mRS	0	0.40	0.2229
NIHSS	14.75	17.50	0.4761
IVH (%Y)	75.0%	70.0%	1
HTN (%Y)	75.0%	70.0%	1
Spot Sign (%Y)	25.0%	30.0%	1
Brain Depth (%‘Deep’)	50.0%	70.0%	0.5804
1.2 Preoperative Care			
Bleed to Evacuation (hours)	11.395	26.94	**0.041**
Anticoagulants (%Y)	50.0%	10.0%	0.1758
Angiographic Event (%Y)	100.0%	90.0%	1
Antiplatlets (%Y)	25.0%	10.0%	0.5055
Pre-Op Volume (mL)	48.9407	72.01	0.224
1.3 Intraoperative Care			
Total Time (hours)	5.275	3.65	0.073
1.4 Postoperative Outcomes			
Post-Op Volume (mL)	17.8853	4.78	**0.0054**
Evacuation (%)	60.4%	94.4%	0.0531
NSICU LOS	8.5	8.50	0.9376
LOS	23	16.10	0.3438
6-month mRS	2.75	3.60	0.2526
Mortality (%Died)	0.0%	10.0%	1

mRS: modified Rankin Scale; NIHSS: NIH Stroke Scale; IVH: intraventricular hemorrhage; HTN: hypertension; NSICU LOS: neurosurgical intensive care unit length of stay; LOS: Length of stay.

**Table 3:** Odds ratio of surgical failure at the completion of an initial EP. A multivariate logistic regression analysis was performed with significant covariates identified previously. Presence of IVH and increased age were found to be associated with an increased likelihood of initial surgical failure. Longer bleed-to-evacuation times were found to be significantly associated with lower odds of surgical failure at the completion of the initial EP.

Failure at End of Initial EP Odds Ratio		
Parameter	OR	95% CI
IVH (Y)	4.349	1.512–12.507
Age (years)	1.049	1.007–1.092
Bleed-to-Evacuation (hours)	0.97	0.945–0.997

## Data Availability

The original contributions presented in the study are included in the article, further inquiries can be directed to the corresponding author.

## Abbreviations

ICH	intracerebral hemorrhage
MIS	minimally invasive surgery
CT	computed tomography
iCT	intraoperative conebeam CT
EP	evacuation period
Angiosuite	angiography suite
SCUBA	Stereotactic Underwater Blood Aspiration
HTN	hypertension
mRS	modified Rankin Score
NIHSS	National Institute of Health Stroke Score
IVH	intraventricular hemorrhage
NSICU	neurosurgical intensive care
LOS	length of stay
ANOVA	analysis of variance
